# Formation and optical properties of metal/10-hydroxybenzo[h]quinolone complexes in the interlayer spaces of magadiite by solid–solid reactions

**DOI:** 10.1098/rsos.171732

**Published:** 2018-05-23

**Authors:** Yifu Zhang, Shengnan Gao, Hanmei Jiang, Qiushi Wang, Yan Cheng, Jiang Zhu, Changgong Meng

**Affiliations:** 1 School of Chemistry, Dalian University of Technology, Dalian 116024, People's Republic of China; 2 College of Chemistry and Chemical Engineering, Liaoning Normal University, Dalian 116029, People's Republic of China

**Keywords:** layered silicates, complexes, 10-hydroxybenzo[h]quinolone, solid–solid reactions, optical properties

## Abstract

Intercalation and *in situ* formation of three fluorescent complexes, Al(III)-, Cr(III)- and Cu(II)-10-hydroxybenzo[h]quinolone (M-HBQ, M = Al, Cr and Cu), in the interlayer spaces of magadiite (mag) were studied by solid–solid reactions between metal ions exchanged mags (M-mag, M = Al, Cr and Cu) and HBQ. Results show that the basal spacings of the intercalated composites increase after the intercalation of HBQ into M-mags. The amount of HBQ in the intercalated compounds is different due to the amount of metal ions and the diversification of coordination ability of metal ions, and the order of the coordination ability of these three metal ions is Cu^2+^ > Cr^3+^ > Al^3+^. The amount of the metal cations in the interlayer of mag is enough for the *in situ* complex formation of M-HBQ complexes. The slight shift of the absorption and luminescence bands of the complexes suggests the different microstructures, including molecular packing of the complexes in the interlayer spaces of mags, resulting that the host–guest interactions are formed. These findings show that the intercalation and *in situ* formation of M-HBQ complexes (M = Al, Cr and Cu) in the interlayer space of mag are successfully achieved in the current work.

## 1. Introduction

In the past decades, intercalation of guest species into layered inorganic solids has attracted great attention from a wide range of scientific and practical viewpoints [1–7]. Intercalation of photoactive species like organic dyes into layered solids has been investigated to understand the nature of host–guest systems and to prepare novel photofunctional supramolecular systems [8], because the characteristics of the photoprocesses are sensitive to the environment in which the photoactive species are located [9]. It has been found that solid–solid reactions are promising ways to intercalate organic guest species into the interlayer spaces of inorganic solids [2,9,10]. Solid–solid reactions are one of the most suitable techniques for intercalated processing due to the facile operation and the possibility to prepare compounds, which are not accessible from solutions, and so on [11,12]. Owing to the advantages of solid–solid reactions, it is worth investigating for constructing novel low-dimensional nanohybrid materials with novel structures and properties by the solid-state intercalation of metal complexes into layered inorganic solids [9,10,13]. The solid–solid intercalation of both cationic and non-ionic species into inorganic solids, such as layered clay minerals, layered zirconium phosphate and zeolites, has been reported so far [2,9,14–17].

**Scheme 1.**
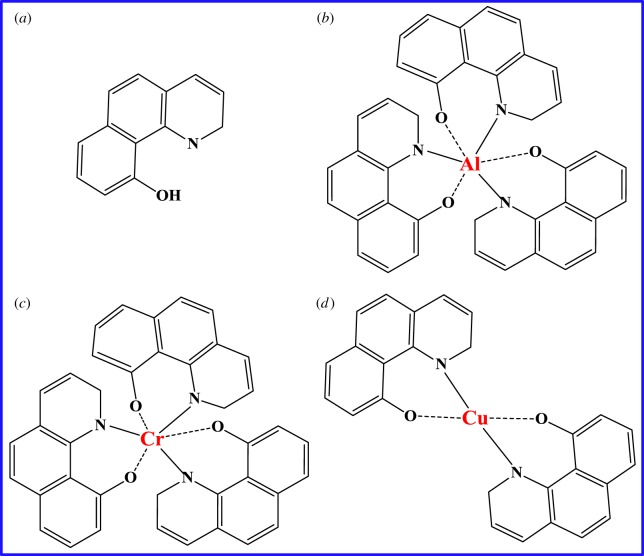
Molecular structure of HBQ and M(HBQ)_n_ (M = Al, Cr, Cu).

Magadiite (Na_2_Si_14_O_29_·*x*H_2_O, abbreviated as mag) was first found in the deposits of Lake Magadi in Kenya and described by Eugster in 1967 [18]. The structure of mag is composed of one or multiple negatively charged sheets of SiO_4_ tetrahedra with abundant silanol-terminated surfaces. Negative charges in the layers of mag are counterbalanced by hydrated cations (Na^+^ or H^+^ *et al.*) in the interlayer spaces [19–22]. Mag has a high cation exchange capacity (CEC) that can be applied to ion exchange, whereby the sodium ions can be replaced by protons, other cations or large quaternary ammonium ions [23–25]. These properties of mag prove it to be a good candidate for the formation of organic–inorganic nanocomposites as the host material. In the previous reports, Makoto Ogawa intercalated tris(2,2′-bipyridine)ruthenium(II) complex [8,26], 1,1′-diethyl-2,2′-cyanine [27], 4-dodecyloxy-4′-(trimethylammoniopentyloxy)azobenzene and 4-(ω-trimethylammoniodecyloxy)-p′-(octyloxy)azobenzene bromide [28], p-[2-(2-hydroxyethyldimethylammonio)ethoxy]azobenzene bromide [29] in the interlayer space of mag. However, these works mainly focused on the intercalation, and their properties were not studied. Recently, the intercalation of inorganic nanoparticles (e.g. ZnO, CuO, CdS and ZnS) into mag has been received increasing attention [30–34]. The intercalation of fluorescers into the layer space of mag has been less reported compared with montmorillonite [9,14,15,35]. In our previous reports, we studied the self-assembly of 8-hydroxyquinoline-Li(I), Al(III) and Cu(II) complexes [36] and the intercalation and *in situ* formation of coordination compounds with ligand 8-hydroxyquinoline-5-sulfonic acid [37] into the interlayer surfaces of mag. Herein, we are interested in extending the previous studies to the intercalation of 10-hydroxybenzo[h]quinolone (abbreviated as HBQ, the molecular structure is shown in scheme 1*a*) into the interlayer space of metal ions exchanged mags (abbreviated as M-mags, M = Al, Cr and Cu). To the best of our knowledge, the intercalation of HBQ into the layered inorganic solids has not been fabricated so far.

HBQ (scheme 1*a*) has been found potential application as a reagent in the synthesis of optical filter agents in photographic emulsion in the previous decades [38,39]. Metal-HBQ and substituted HBQ complexes have attracted great attention owing to their useful luminescence properties especially in the use for organic light-emitting devices (OLEDs) [40,41]. The design and fabrication of efficient OLEDs based on organic materials have been areas of active research because of their important applications in the large area display technology. However, the formation and properties of metal-HBQ in the confined regions of layered silicates like mag have not been reported until now. In the present work, the intercalation and *in situ* complex formation of HBQ with the interlayer metal cations (Al^3+^, Cr^3+^ and Cu^2+^) were investigated to form coordination complexes (scheme 1*b*–*d*) in the interlayer spaces of mag by the solid–solid reactions.

## 2. Material and methods

### 2.1. Materials

**Scheme 2.**
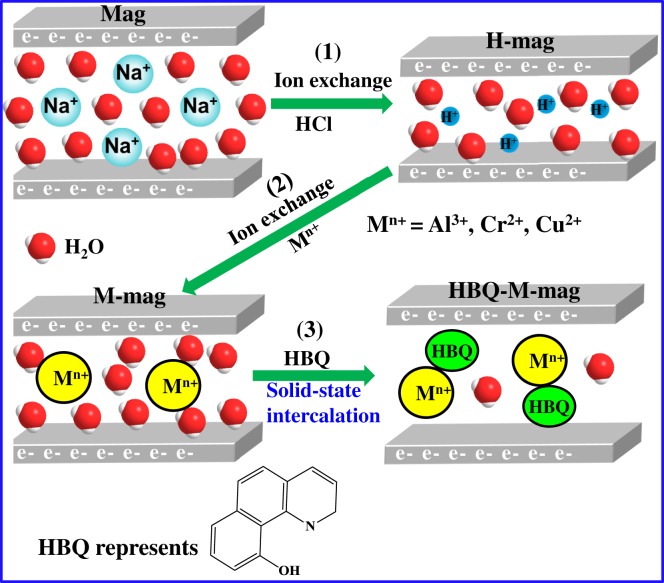
A schematic illustration of preparation of M(HBQ)_n_ (M = Al, Cr, Cu) in the interlayer space of mags and proposed structures of mag, H-mag, M-mag and HBQ-M-mag.

Sodium hydroxide (NaOH), hydrochloric acid (HCl) aluminium sulphate (Al_2_(SO_4_)_3_·18H_2_O), chromium chloride hexahydrate (CrCl_3_·6H_2_O), copper sulphate pentahydrate (CuSO_4_·5H_2_O), 10-hydroxybenzo[h]quinoline (C_13_H_9_NO, the structure is shown in scheme 1*a*, abbreviated as HBQ) and silica gel (30 wt% Aldrich), were purchased from Sinopharm Chemical Reagent Co., Ltd. All chemicals were of analytical grade and used without any further purification. Scheme 2 schematically illustrates the synthetic process of HBQ-M-mags (M = Al, Cr and Cu) in the interlayer spaces of mags and the detailed process is described in the following sections.

### 2.2. Synthesis of mag and H-mag

Sodium magadiite (abbreviated as mag) was hydrothermally synthesized based on our previous report [36]. In a typical synthesis, mixtures of silica gel and NaOH with a molar ratio SiO_2_ : NaOH : H_2_O = 9 : 3 : 162 were sealed in a Teflon-lined autoclave and hydrothermally treated at 150°C for 48 h. After reaction, suspension was filtered and washed carefully with distilled water to remove excess NaOH, and dried at 80°C for 24 h. The empirical chemical formula of the obtained mag can be expressed as Na_2_Si_14_O_29_·9H_2_O. Cation exchange capacity (CEC) of mag was 200 meq/100 g [25]. H^+^ exchanged mag (abbreviated as H-mag) was synthesized by adding mag to HCl (0.1 M) until pH is about 2 with magnetic stirring for 24 h. When the ion exchange reactions were finished, the products were obtained with a centrifugal separator and washed with deionized water several times until a negative AgNO_3_ test was achieved.

### 2.3. Synthesis of M-mags (M = Al, Cr, Cu)

Metal ions (Al^3+^, Cr^3+^ and Cu^2+^) exchanged mags (abbreviated as M-mags, M = Al, Cr and Cu) were synthesized by the second ion exchange step. In detail, 1 M fresh aqueous solution of Al^3+^, Cr^3+^ and Cu^2+^ were separately mixed with H-mag and these mixtures were magnetically stirred at room temperature. After 24 h, the resulting products were obtained with a centrifugal separator and washed with deionized water several times until a negative AgNO_3_ test was achieved. The as-obtained samples were named as Al-mag, Cr-mag and Cu-mag, respectively.

### 2.4. Intercalation of HBQ into the interlayer spaces of M-mags (M = Al, Cr, Cu)

Intercalation of HBQ into the interlayer spaces of M-mags (M = Al, Cr, Cu) was prepared by solid–solid reactions reported by Makoto Ogawa [9,10,14]. HBQ was mixed with M-mags and ground manually using an agate mortar and a pestle at ambient environment for 15 min. Molar ratios of HBQ to interlayer cations were 3 : 1, 3 : 1 and 2 : 1 for Al-, Cr- and Cu-mags, respectively. After solid–solid reactions, intercalated compounds were washed with n-hexane and ethanol several times and dried at 60°C for 24 h. The synthesized products were marked as HBQ-Al-mag, HBQ-Cr-mag and HBQ-Cu-mag, respectively.

### 2.5. Materials characterizations

Powder X-ray diffraction (XRD) was collected on a Panalytical X'Pert Powder diffractometer using monochromatic Cu K*α* radiation. The amounts of exchange cations were determined by an energy-dispersive X-ray spectrometer (EDS) attached to a scanning electron microscope (SEM, QUANTA450). X-ray photoelectron spectroscopy (XPS) was used to investigate the surface composition of the products performed on ESCALAB250Xi, Thermo Fisher Scientific. Fourier-transform infrared (FTIR) spectra of the samples were recorded by KBr disc method on a Niole Avatar 360 FTIR spectrometer (USA) over the spectral region of 400–4000 cm^−1^. The morphology and dimensions of the products were observed by field emission scanning electron microscopy (FE-SEM, NOVA NanoSEM 450, FEI). Samples for FE-SEM observation were gold-sputtered in order to get better morphology of the surface. Diffuse reflectance spectra (UV-Vis) of the solid samples were collected on an American HP-8453 scanning spectrophotometer using an integrated sphere. Photoluminescence spectra (PL) were characterized on a standard Jasco FP-6500 spectrofluorophotometer with the excitation at 395 nm. Inverted fluorescence microscope was tested on a Japan Olympus (IX71+DP71) with the excitation at 360 nm.

## 3. Results and discussion

**Figure 1.**
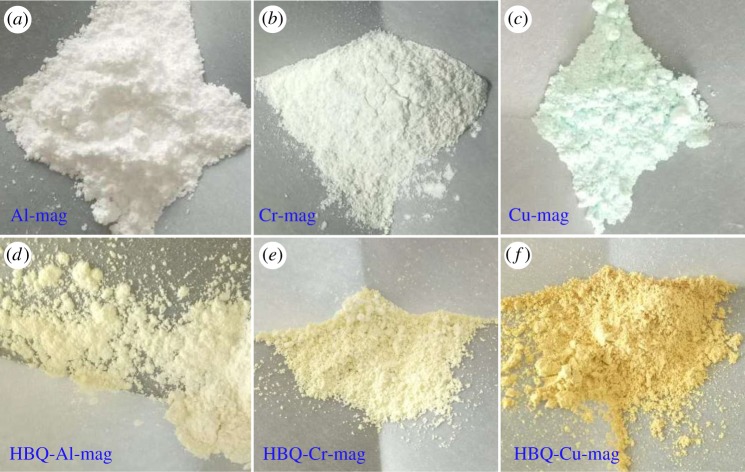
Colours of M-mags and HBQ-M-mags (M = Al, Cr and Cu).

Scheme 2 shows the synthetic process of HBQ-M-mags (M = Al, Cr and Cu) in the interlayer spaces of mags, which mainly comprise four steps including synthesis of mag, two step ion exchanges (H-mag and M-mag) and solid–solid reactions. Mag and H-mag are white powders. After the ion exchange with metal ions and the solid–solid reaction with HBQ, the colours of M-mags and HBQ-M-mags are shown in figure 1. Al-mag, Cr-mag and Cu-mag exhibit the white (figure 1*a*), light green (figure 1*b*) and light blue colours (figure 1*c*) in agreement with the colour of the corresponding ions, which may suggest that the hydrated M-mags are synthesized. After the solid–solid reaction between M-mags and HBQ, the colours of the hydrated M-mags change from white to yellow (figure 1*d*–*f*). The colours of HBQ-Al-mag, HBQ-Cr-mag and HBQ-Cu-mag are light yellow, light yellow and yellow, respectively. The change in the colours of M-mags after the intercalated HBQ suggests the change in the coordination state of hydrated metal interlayer cation (Al^3+^, Cr^3+^ or Cu^2+^) [9].

**Figure 2.**
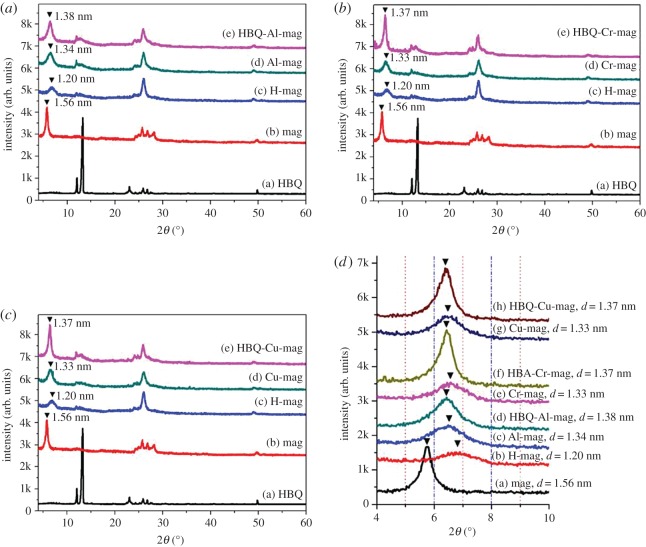
XRD patterns of HBQ, mag, H-mag, M-mags and HBQ-M-mags (M = Al, Cr, Cu): (*a*) HBQ-Al-mag series; (*b*) HBQ-Cr-mag series; (*c*) HBQ-Cu-mag series; (*d*) Comparison of the *d* values of various mags.

**Figure 3.**
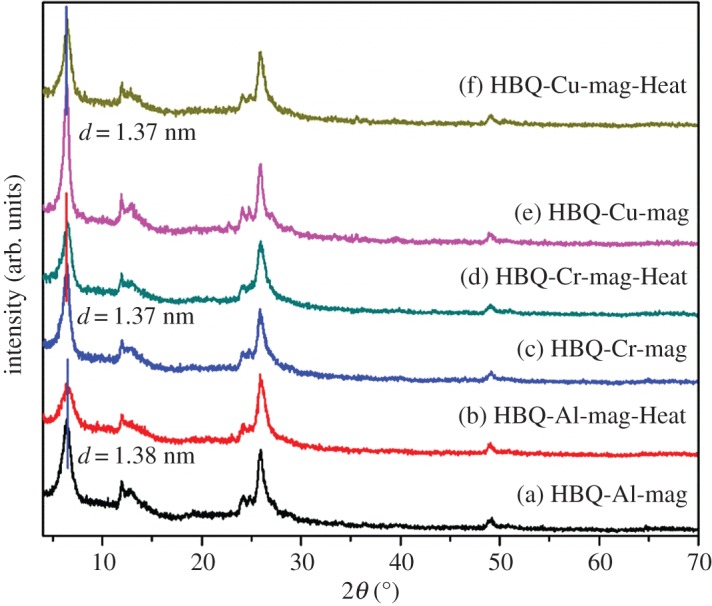
XRD patterns of HBQ-M-mags and their corresponding samples obtained by heat treatment.

Figure 2 shows XRD patterns of HBQ, mag, H-mag, M-mags and HBQ-M-mags (M = Al, Cr, Cu) and the results indicate that M-HBQ are successfully intercalated into the interlayer spaces of mag. Figure 2*a*–*c* respectively depict XRD patterns of HBQ-Al-mag series, HBQ-Cr-mag series, HBQ-Cu-mag series, and figure 2*d* shows the comparison of the *d* values of various mags. From XRD patterns (figure 2*a*–*c*), all of M-mags and HBQ-M-mags exhibit the phase of H-mag, which is very consistent with our designed synthesis. To clearly reveal the change of the basal spacing, XRD patterns (2*θ* = 4–10°) of M-mags and HBQ-M-mags are summarized in figure 2*d*. The diffraction peaks (001) of mag and H-mag, are observed at 1.56 nm and 1.20 nm, respectively. This decrease of the basal spacing proves the formation of H-mag after the ion exchange with H^+^. The basal spacings of Al-mag, Cr-mag and Cu-mag appear at 1.34, 1.33 and 1.33 nm, respectively. Compared with the basal spacing of H-mag (1.20 nm), these basal spacings increase after the second ion exchange with Al^3+^, Cr^3+^ or Cu^2+^, which indicates the successful preparation of M-mags. The interlayer spaces of M-mags are hydrated irrespective of the composition and the amounts of water that are about 4–6 wt% [42]. After M-mags are reacted with HBQ by solid-state intercalation, the basal spacings increase to 1.38, 1.37 and 1.37 nm for HBQ-Al-mag, HBQ-Cr-mag and HBQ-Cu-mag, respectively. XRD patterns of the products show no reflections due to HBQ crystal, suggesting that the ligand HBQ is intercalated into the interlayer spaces of M-mags. The basal spacing of (001) reflection shifts to larger *d* values when HBQ is intercalated into M-mags and the reflections characteristic to HBQ are not observed. It is noteworthy that no segregation phenomenon is observed in XRD patterns because of the stability of M-HBQ complexes formed in the interlayer space of mag. The changes in the colours and XRD results of the products support the solid-state intercalation and *in situ* complex formation of M-HBQ complexes (M = Al, Cr, Cu) in the interlayer space of mag. Because the interlayer exchangeable cations of mags are surrounded by water molecules at ambient conditions [3,43], the increase in the basal spacings of M-HBQ complexes is caused by the intercalation of HBQ though ligand displacement reactions between the adsorbed H_2_O and HBQ molecules. The gallery heights are determined by subtracting the thickness of the silicate layer (1.12 nm) [44] from the observed basal spacings to be 0.26 nm for HBQ-Al-mag, 0.25 nm for HBQ-Cr-mag and 0.25 nm for HBQ-Cu-mag. Taking the gallery heights into account, it is worthwhile to mention that M-HBQ complexes form a monolayer arrangement in between the silicate layers, which is very consistent with the results reported for coordination complexes-montmorillonite hybrids [9,14,45]. By the way, free M-HBQ complexes (M = Al, Cr, Cu) cannot be directly intercalated in the interlayer spaces of mag because of their molecular sizes and neutral charges [9]. Figure 3 shows XRD patterns of HBQ-M-mags (M = Al, Cr, Cu) and their corresponding samples obtained by the heat treatment at 200°C for 2 h. The XRD patterns and basal spacings of HBQ-M-mags-Heat are similar with those of HBQ-M-mags, further supporting the intercalation of M-HBQ complexes in the interlayer space of mags.

**Figure 4.**
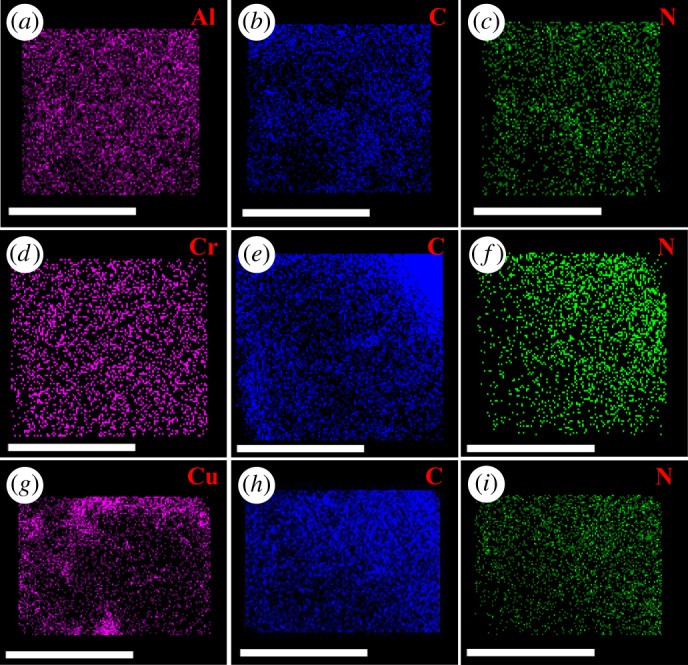
Elemental mapping images of Al, Cr, Cu, C and N: (*a*–*c*) HBQ-Al-mag; (*d*–*f*) HBQ-Cr-mag; (*g*–*i*) HBQ-Cu-mag. The scale bar is 10 µm.

The composition of HBQ-M-mags (M = Al, Cr, Cu) was further confirmed by EDS, elemental mapping, XPS and TG/DTA measurements. Figure S1 in the electronic supplementary material shows EDS spectra of HBQ-Al-mag, HBQ-Cr-mag and HBQ-Cu-mag. The C, O, Si and M (M = Al, Cr, Cu) elements are distinctly seen in these three samples. The peak of the element N is not observed because this element is very little in the intercalated composites. Elemental distribution was provided by elemental mapping, as shown in figure S2 in the electronic supplementary material and figure 4. The elemental mapping images reveal that HBQ-M-mags contain the same elements, with the results of EDS and the N element, which is very little in the samples, also observed. Elemental mapping images show that all elements are homogeneously distributed in HBQ-M-mags.

**Figure 5.**
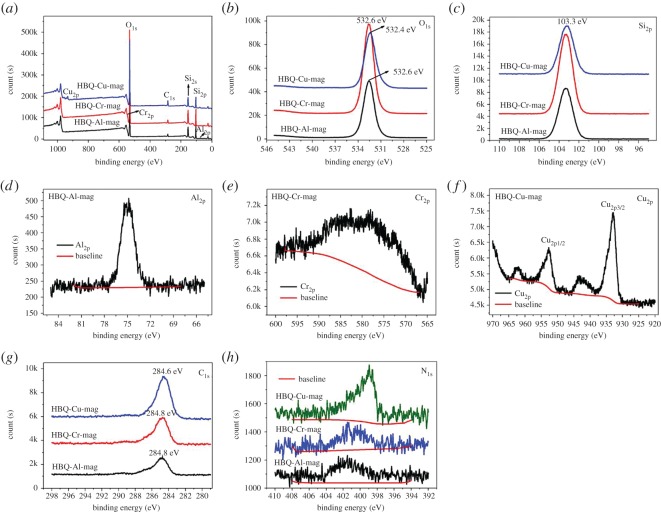
XPS spectra of HBQ-M-mags (M = Al, Cr and Cu): (*a*) full spectra; (*b*) core-level spectra of O_1s_; (*c*) core-level spectra of Si_2p_; (*d*) core-level spectrum of Al_2p_; (*e*) core-level spectrum of Cr_2p_; (*f*) core-level spectrum of Cu_2p_; (*g*) core-level spectra of C_1s_; (*h*) core-level spectra of N_1s_.

The information on the composition and chemical/electronic state of various elements in the products are provided by XPS, as shown in figure 5 and table 1. Figure 5*a* depicts the full XPS spectra of HBQ-M-mags (M = Al, Cr, Cu) and the elements Si, O and C are seen in these three samples, and elements Al, Cr and Cu are also observed in the corresponding samples. In full XPS spectra, element N is not observed owing to its little amount. However, when the core-level spectra are used to determine N element, N element is clearly detected as shown in figure 5*h*. These findings are well consistent with EDS spectra and elemental mappings of HBQ-M-mags as shown in figure 4 and figures S1 and S2 in the electronic supplementary material. The core-level spectra of O_1s_ and Si_2p_ are depicted in figure 5*b* and *c*. The binding energies of O_1s_ in HBQ-Al-mag, HBQ-Cr-mag and HBQ-Cu-mag locate at 532.6, 532.6 and 532.4 eV, and the binding energies of Si_2p_ in HBQ-M-mags centre at 103.3 eV. These peaks are from the mag [24]. Figure 5*d*, *e* and *f* respectively show the core-level spectrum of Al_2p_, Cr_2p_ and Cu_2p_, and these peaks exhibit low intensity owing to their low content. The Cu_2p_ splits off into two peaks including Cu_2p1/2_ (932.9 eV) and Cu_2p3/2_ (952.8 eV). These binding energies indicate that the oxidation state of copper is +2. The results are also in a good agreement with the data previously reported [46]. Figure 5*g* shows the core-level spectra of C_1s_ in HBQ-M-mags. The binding energies of C_1s_ locate at 284.8, 284.8 and 284.6 eV in HBQ-Al-mag, HBQ-Cr-mag and HBQ-Cu-mag, respectively, which may be caused by the difference of the constrained geometry of the host, and the arrangements of the complexes in the interlayer spaces as well as the increased intermolecular interaction between adjacent molecules in solid state [9,14,47,48].

**Table 1.** Summary of chemical content of HBQ-M-mags (M = Al, Cr and Cu) by XPS.

	samples			
	content (atom %) from XPS			
elements	HBQ-Al-mag	HBQ-Cr-mag	HBQ-Cu-mag	HBQ
M^a^	1.98	1.25	1.36	
O	61.40	61.54	57.03	
Si	29.30	30.02	26.82	
C	6.75	6.66	13.80	
N	0.57	0.54	0.99	
C/M in the sample^b^	3.41	5.33	10.21	
C/M in theory^c^	39	39	26	
M/N in the sample^d^	3.47	2.31	1.37	
M/N in theory^e^	0.33	0.33	0.50	
C/N	11.84	12.33	13.94	13

^a^M = Al, Cr and Cu.

^b^C/M represents the mole ratio of C/M.

^c^C/M in theory represents the mole ratio of C/M in HBQ-M as the molecular structure shown in scheme 1.

^d^M/N represents the mole ratio of M/N.

^e^M/N in theory represents the mole ratio of M/N in HBQ-M as the molecular structure shown in scheme 1 (M = Al, Cr and Cu).

Table 1 summarizes chemical content of HBQ-M-mags by XPS and the corresponding calculations. From C/M in the sample and C/M in theory, the amount of the metal cations (Al^3+^, Cr^3+^ or Cu^2+^) in the interlayer of mag is fair enough for the *in situ* complex formation of M-HBQ complexes; that is, these samples contain residual metal cations after the solid–solid reactions between M-mags and HBQ. The same results can also be provided by M/N in the sample and M/N in theory. The above results further suggest the formation of M-HBQ complexes in the interlayer space of mag. The order of the coordination ability of these three metal ions is Cu^2+^ > Cr^3+^ > Al^3+^. As depicted in table 1, the ratio of C/N in HBQ-M-mags is close to the corresponding values in HBQ. Therefore, XPS results further indicate the successful intercalation and formation of M-8HqS complexes (M = Al, Ca and Zn) in the interlayer space of mag.

To calculate the amount of the intercalated HBQ in HBQ-M-mags (M = Al, Cr and Cu), the heat method in the air atmosphere was used to study the amount of intercalated HBQ. M-mags and HBQ-M-mags were calcined at 500°C for 2 h in air, and the corresponding results are shown in table 2. The amounts of HBQ measure 7.12, 11.50 and 9.17 wt% in HBQ-Al-mag, HBQ-Cr-mag and HBQ-Cu-mag, respectively. The difference in the amount of HBQ in the intercalated compounds that is caused by the coordination ability of these three metal ions (Al^3+^, Cr^3+^ and Cu^2+^) is diverse in agreement with XPS results. All the above results confirm the intercalation of HBQ into the interlayer spaces of M-mags.

**Table 2.** Weight loss of HBQ from the calcination of M-mags and HBQ-M-mags (M = Al, Cr and Cu) at 500°C for 2 h in air.

samples	m_1_ (g)^a^	m_2_ (g)^b^	weight loss (wt%)^c^	amount of HBQ (wt%)^d^
Al-mag	0.1998	0.1891	5.36	
HBQ-Al-mag	0.2028	0.1775	12.48	7.12
Cr-mag	0.2009	0.1885	6.17	
HBQ-Cr-mag	0.2043	0.1682	17.67	11.50
Cu-mag	0.2003	0.1908	4.74	
HBQ-Cu-mag	0.2051	0.1753	14.53	9.79

^a^The weight before the calcination.

^b^The weight after the calcination at 500°C for 2 h in air.

^c^The weight loss (wt%) was calculated by the equation: (m_1_ − m_2_)/m_1_ * 100%.

^d^The amount of HBQ (wt%) was calculated by the weight loss of HBQ-M-mags deducting from the mass loss of M-mags (M = Al, Ca and Zn).

**Figure 6.**
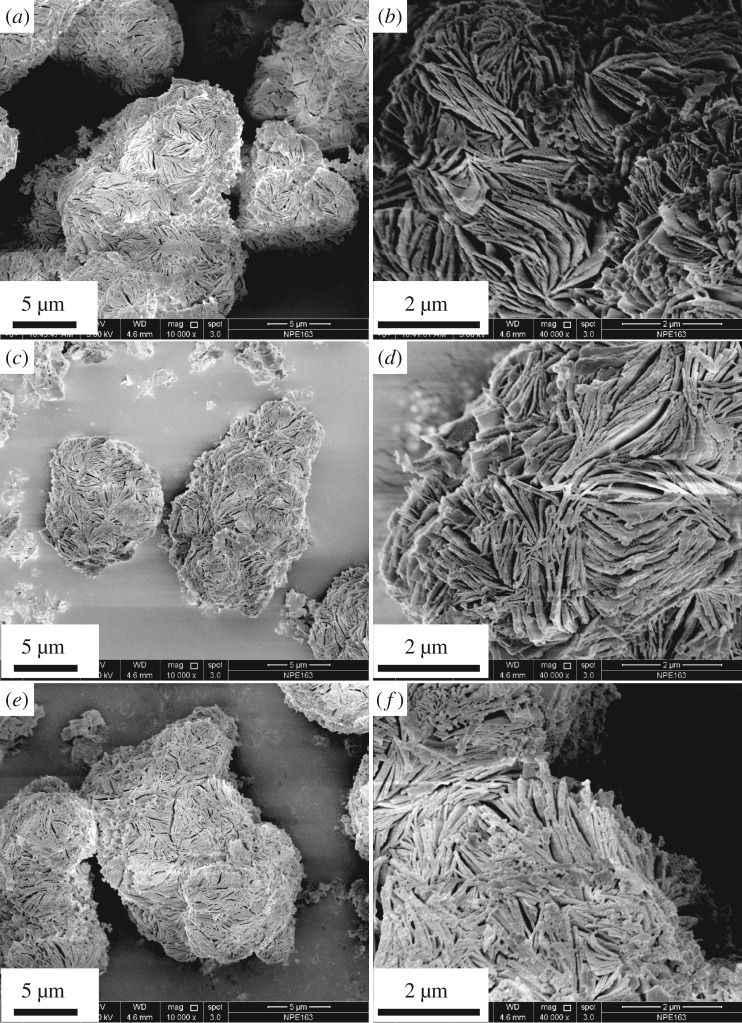
FE-SEM images of (*a*,*b*) Al-mag, (*c*,*d*) Cr-mag and (*e*,*f*) Cu-mag.

**Figure 7.**
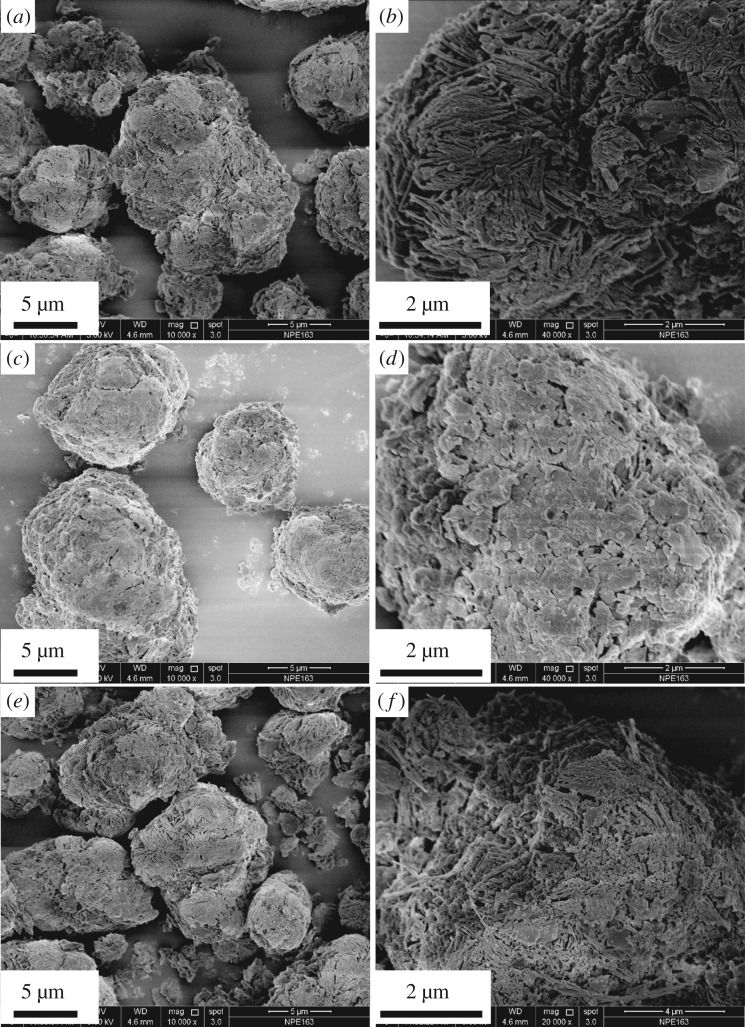
FE-SEM images of (*a*,*b*) HBQ-Al-mag, (*c*,*d*) HBQ-Cr-mag and (*e*,*f*) HBQ-Cu-mag.

Morphologies of mag, M-mags and HBQ-M-mags (M = Al, Cr and Cu) were observed by FE-SEM. Figure S3 in the electronic supplementary material and figures 6 and 7 show FE-SEM images of mag, Al-mag, Cr-mag, Cu-mag, HBQ-Al-mag, HBQ-Cr-mag and HBQ-Cu-mag, respectively. As shown in figure 6, most of the platelets in Al-mag, Cr-mag and Cu-mag are well preserved without severe destruction compared with original mag (figure S3 in the electronic supplementary material). Figure 7 depicts FE-SEM images of HBQ-Al-mag, HBQ-Cr-mag and HBQ-Cu-mag. It is observed that HBQ-Al-mag, HBQ-Cr-mag and HBQ-Cu-mag kept the original platelets. However, the platelets became disordered to some extent. The morphology of platelets of various intercalated mags is slightly destroyed, which is caused by the grind process [36].

**Figure 8.**
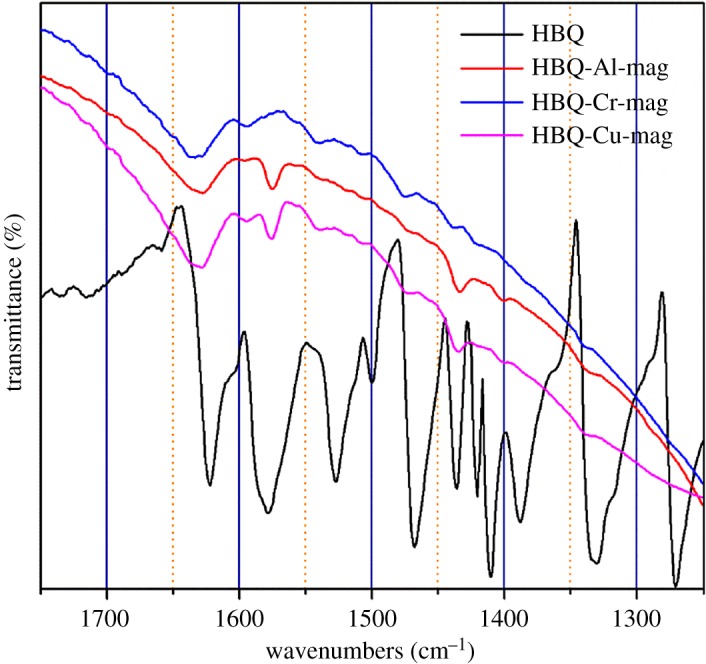
FTIR spectra of HBQ and HBQ-M-mags (M = Al, Cr and Cu).

The *in situ* complex formation of Al, Cr and Cu interlayer cations and the ligand HBQ was further determined by FTIR, UV-Vis and PL spectroscopies. Figure S4*a*–*c* in the electronic supplementary material shows FTIR spectra of HBQ, mag, H-mag, M-mags and HBQ-M-mags (M = Al, Cr and Cu). Comparing these FTIR spectra, their main difference is in the range from 1700 to 1200 cm^−1^ due to the overlap between HBQ and mag in the other region. Figure 8 and table 3 summarize the FTIR spectra of HBQ and HBQ-M-mags in 1700–1200 cm^−1^, and these FTIR peaks are assigned to HBQ [40,41]. Some FTIR peaks of HBQ are not seen in figure 8 because the amount of HBQ in HBQ-M-mags is relatively less. The bands of intercalated products in this region assigned to HBQ are slightly shifted when compared with those observed for free HBQ molecule, supporting the coordination between HBQ and metal interlayer cations in mag [9,49]. FTIR spectra of the intercalated composites do not show any additional absorption bands due to decomposed species, suggesting no decomposition of HBQ molecule. Therefore, the results suggest the formation of M-HBQ complexes in the interlayer space of mag. Because the homogeneous mixture of HBQ or M-HBQ complexes in the hybrids may exhibit the shifts in FTIR spectra [9], the further characterizations of UV-Vis and PL spectroscopies were also used to confirm the *in situ* complex formation of the M-HBQ complexes in the interlayer space of mags.

**Table 3.** Wavenumbers (cm^−1^) of FTIR band of HBQ and HBQ-M-mags (M = Al, Cr and Cu) and their assignments.

assignments	HBQ	HBQ-Al-mag	HBQ-Cr-mag	HBQ-Cu-mag
ring stretching	1622	1627	1628	1629
ring stretching	1578	1574	1575	1574
ring stretching	1527	1540	1540	1541
ring stretching	1500	1506	1506	1508
ring stretching	1468	1474	1473	1475
ring stretching	1436	1435	1435	1439
CH bending	1421	—	—	1421
CH bending	1410	1401	1402	—
CH bending	1388	—	—	—
ring stretching	1330	1339	1337	1339
C-N stretching	1270	—	—	—

**Figure 9.**
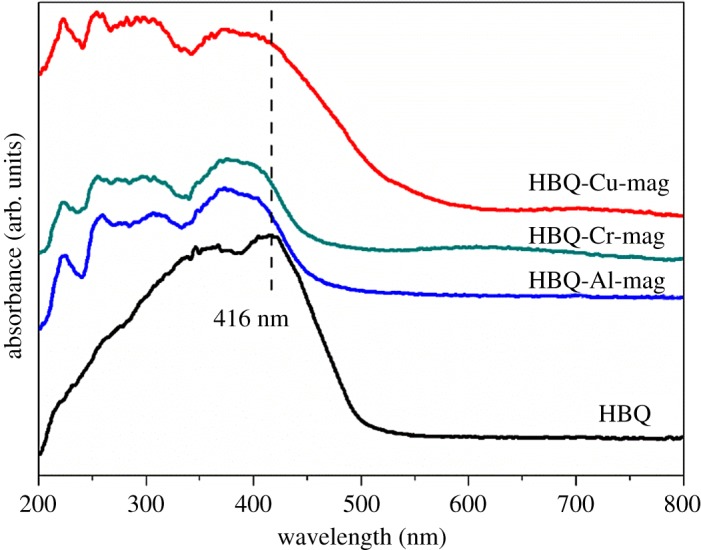
UV-Vis spectra of HBQ, HBQ-Al-mag, HBQ-Cr-mag and HBQ-Cu-mag.

**Figure 10.**
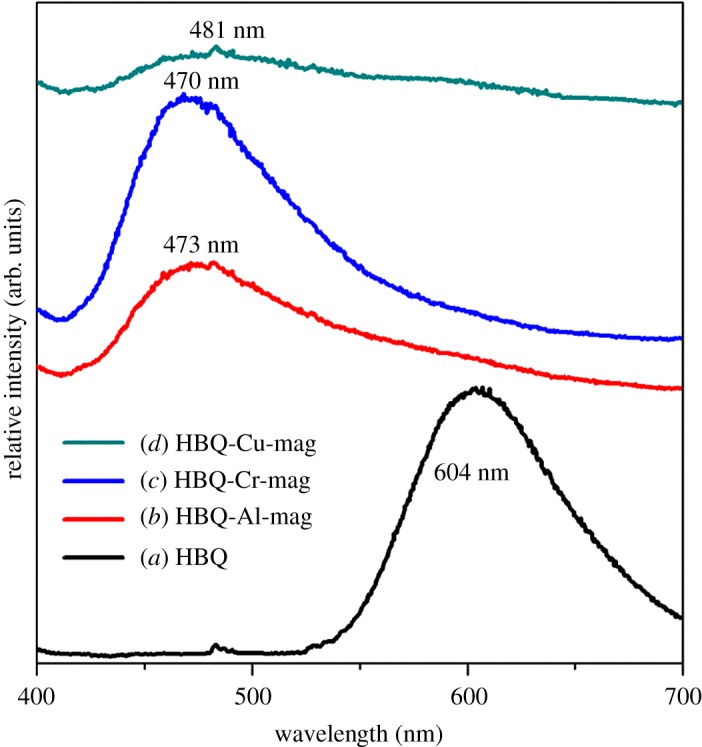
PL spectra of (*a*) HBQ, (*b*) HBQ-Al-mag, (*c*) HBQ-Cr-mag and (*d*) HBQ-Cu-mag.

Figure 9 shows UV-Vis spectra of HBQ, HBQ-Al-mag, HBQ-Cr-mag and HBQ-Cu-mag. In the solid state, HBQ exhibits a broad peak at 416 nm in agreement with the previous report [50]. After intercalation HBQ into M-mags (M = Al, Cr and Cu), this absorption band shifts to the low wavelength. Blue shift suggests the formation of M-HBQ complexes in the interlayer spaces of mag [9,15]. PL spectra of HBQ, M-HBQ and HBQ-M-mags (M = Al, Cr and Cu) are shown in figure 10. As shown in figure 10*a*, the maximum emission band due to the π to π* transition of HBQ in the solid state is located at around 604 nm, which is consistent with previous report [39,50]. After intercalating HBQ into M-mags, as depicted in figure 10*b*–*d*, PL bands of HBQ-Al-mag, HBQ-Cr-mag and HBQ-Cu-mag are located at 473, 470 and 481 nm, respectively. The blue-shifted fluorescence further supports the formation of M-HBQ complexes in the interlayer spaces of mag. In neutral water, HBQ exhibits a dominant fluorescence band at 585 nm, which is tentatively ascribed to the proton transfer tautomer emission, which seems to be reasonable due to the proposed ultrafast rate of excited-state proton transfer for HBQ in both aprotic and protic non-aqueous solutions [51]. This fluorescence band can be adjusted by the acidity of solution. The variation of the luminescence bands is thought to be attributed to the host–guest interactions, though the details are still difficult to illustrate [14]. The PL bands observed in figure 10 for HBQ-Al-mag (473 nm), HBQ-Cr-mag (470 nm) and HBQ-Cu-mag (481 nm) can be attributed to M-HBQ complexes formed in the interlayer spaces of mag. The blue shift of the emission bands of M-HBQ complexes in mag may be potentially due to the constrained geometry of the host, and the arrangements of the complexes in the interlayer spaces as well as the increased intermolecular interaction between adjacent molecules in the solid state [9,14]. These observations uphold the *in situ* complex formation of M-HBQ complexes in the interlayer spaces of mag. Therefore, the molecular structure and packing of M-HBQ complexes formed in the mag differently to make the tiny difference in the wavelength of PL spectra [36]. Inverted fluorescence microscopic images of HBQ-Al-mag, HBQ-Cr-mag and HBQ-Cu-mag were detected under 360 nm UV light irradiation, as shown in figure S5 in the electronic supplementary material. As clearly demonstrated by fluorescence microscope images, HBQ-Al-mag, HBQ-Cr-mag and HBQ-Cu-mag presented yellow fluorescence. On the basis of the above analyses, the intercalation and *in situ* formation of M-HBQ complexes (M = Al, Cr and Cu) in the interlayer space of mag were successfully achieved. The mechanism of the solid-state intercalation has not been well established, but the process may be envisaged as resulting from the tendency of the reaction of a neutral organic molecule and hydrated cations in the interlayer space. According to the states of reactants, the mechanism of HBQ intercalated M-mags can be assigned to the ion–dipole intercalation and coordination [36,52], which are responsible for the adsorption of many non-ionic organic compounds such as amides, alcohols, amines, and ethers [53]. The preparation of M-HBQ in restricted geometries may lead to novel microstructures and physico-chemical properties. The solid-state intercalation along with the *in situ* complex formation of M-HBQ in the interlayer spaces of mag is an effective method to prepare M-HBQ/layered silicate composites. Further studies on the synthesis of intercalated compounds using other ligands with various cations and layered solids are now underway in order to construct layered nanohybrid materials with controlled microstructures and functions.

## 4. Conclusion

We demonstrated that the ligand HBQ was intercalated into the interlayer spaces of M-mags (M = Al, Cr and Cu) by solid–solid reactions at room temperature. After the intercalation of HBQ into M-mags, the basal spacings of the intercalated composites increase. The amount of HBQ in the intercalated compounds is different due to the amount of metal ions and the diversification of coordination ability of metal ions. The amount of the metal cations (Al^3+^, Cr^3+^ and Cu^2+^) in the interlayer of mag is enough for the *in situ* complex formation of M-HBQ complexes. The order of the coordination ability of these three metal ions is Cu^2+^ > Cr^3+^ > Al^3+^. The slight shift of the absorption and luminescence bands of the complexes suggests the different microstructures including molecular packing of the complexes in the interlayer spaces of mags, resulting that the host–guest interactions are formed. Owing to the user- and environment-friendly nature of the solid–solid reactions, the present reactions and the products are useful to practical applications. The studies on solid–solid reaction and *in situ* complex formation of complexes using other ligands or mixed ligands are worth investigating to construct novel nanohybrid materials with precisely controlled properties.

## Supplementary Material

Supplementary Figures

## Data accessibility

The datasets supporting this article have been uploaded as part of the electronic supplementary material.
