# Supplementary material for: Formation and optical properties of metal/10-hydroxybenzo[h]quinolone complexes in the interlayer spaces of magadiite by solid–solid reactions
Source: R Soc Open Sci. 2018 May 23;5(5):171732. doi: 10.1098/rsos.171732 (PMC5990806; doi:10.1098/rsos.171732)
Supplement: Supplementary Figures [file rsos171732supp1.pdf]

Electronic supplementary material

Figure S1

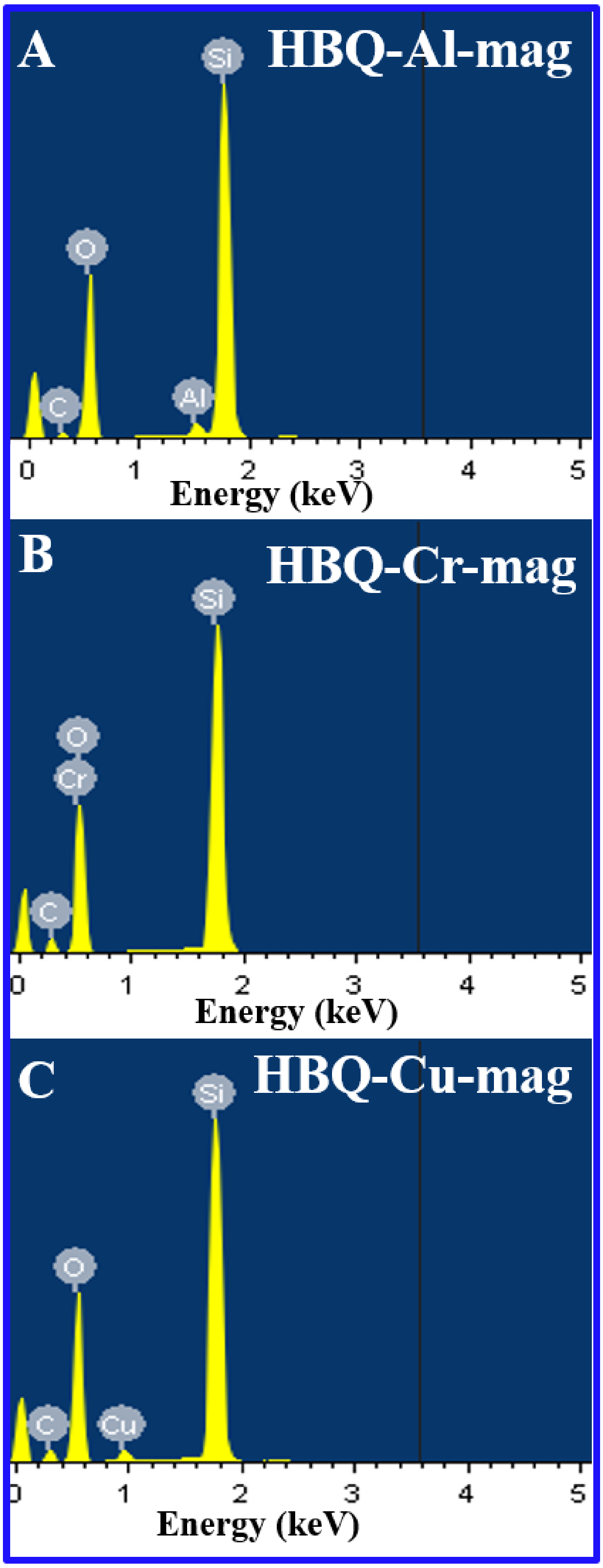

Figure S1. EDS spectra of HBQ-Al-mag (A), HBQ-Cr-mag (B) and HBQ-Cu-mag (C).

Figure S2

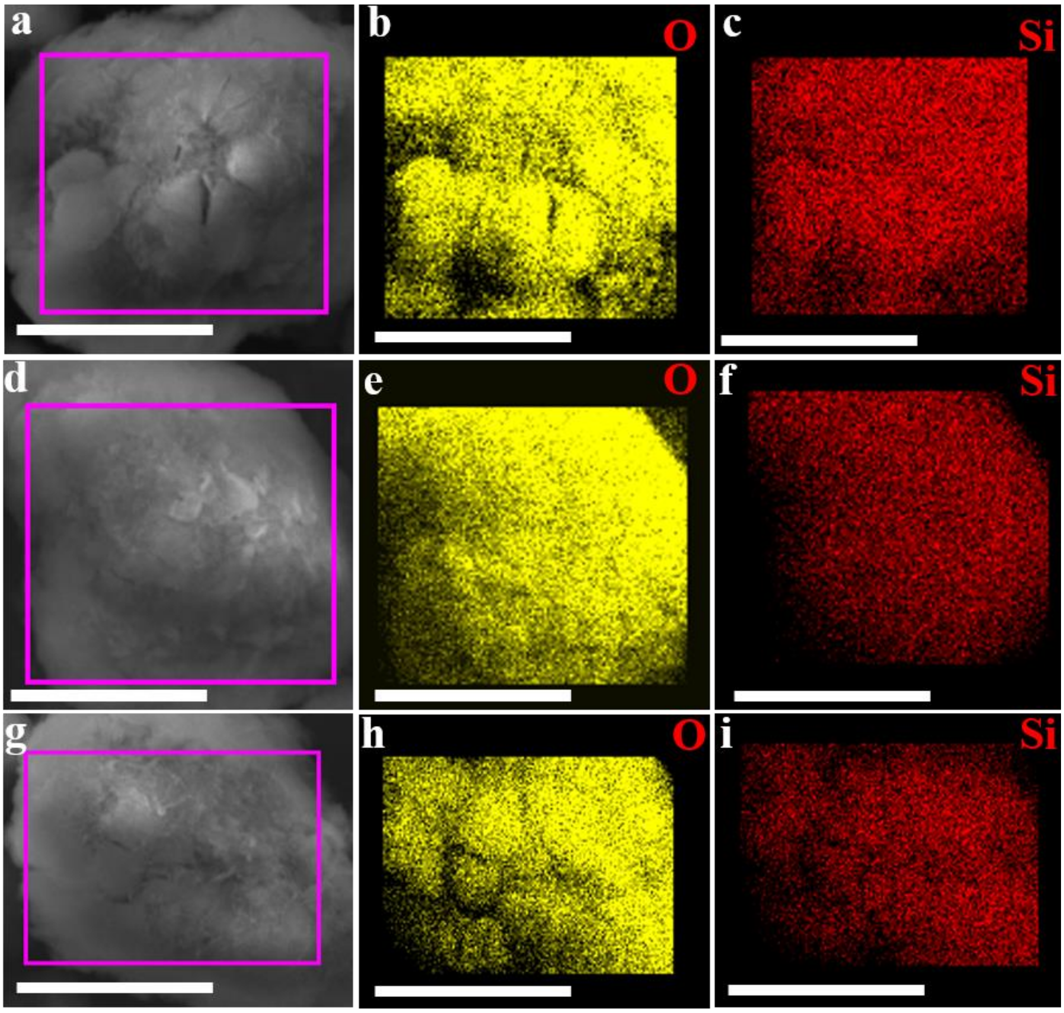

Figure S2. SEM images and their corresponding elemental mapping images of O and Si: (a-c) HBQ-Al-mag; (d-f) HBQ-Cr-mag; (g-i) HBQ-Cu-mag. The scale bar was 10 μm.

Figure S3

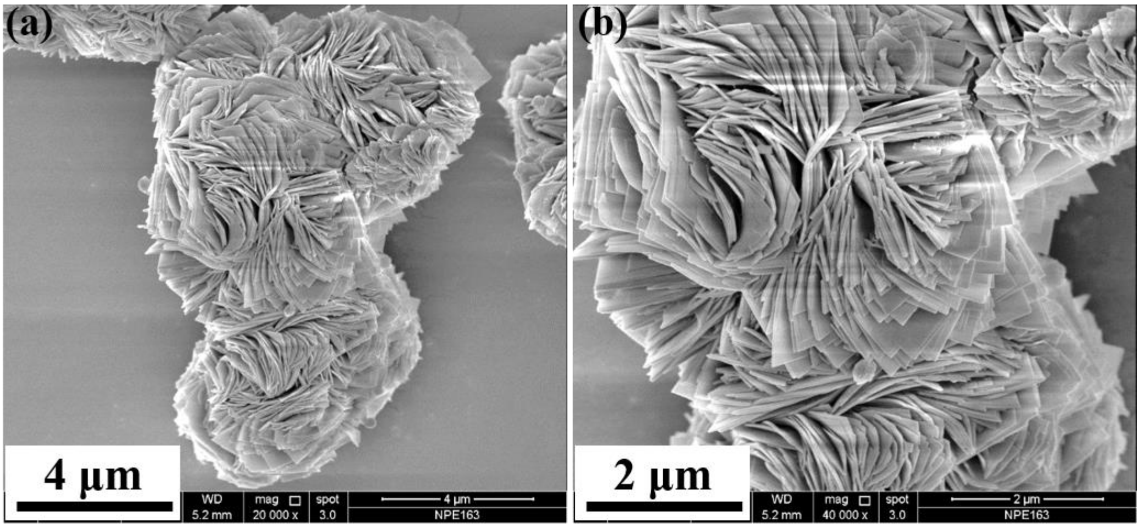

Figure S3. FE-SEM images of the starting mag.

Figure S4

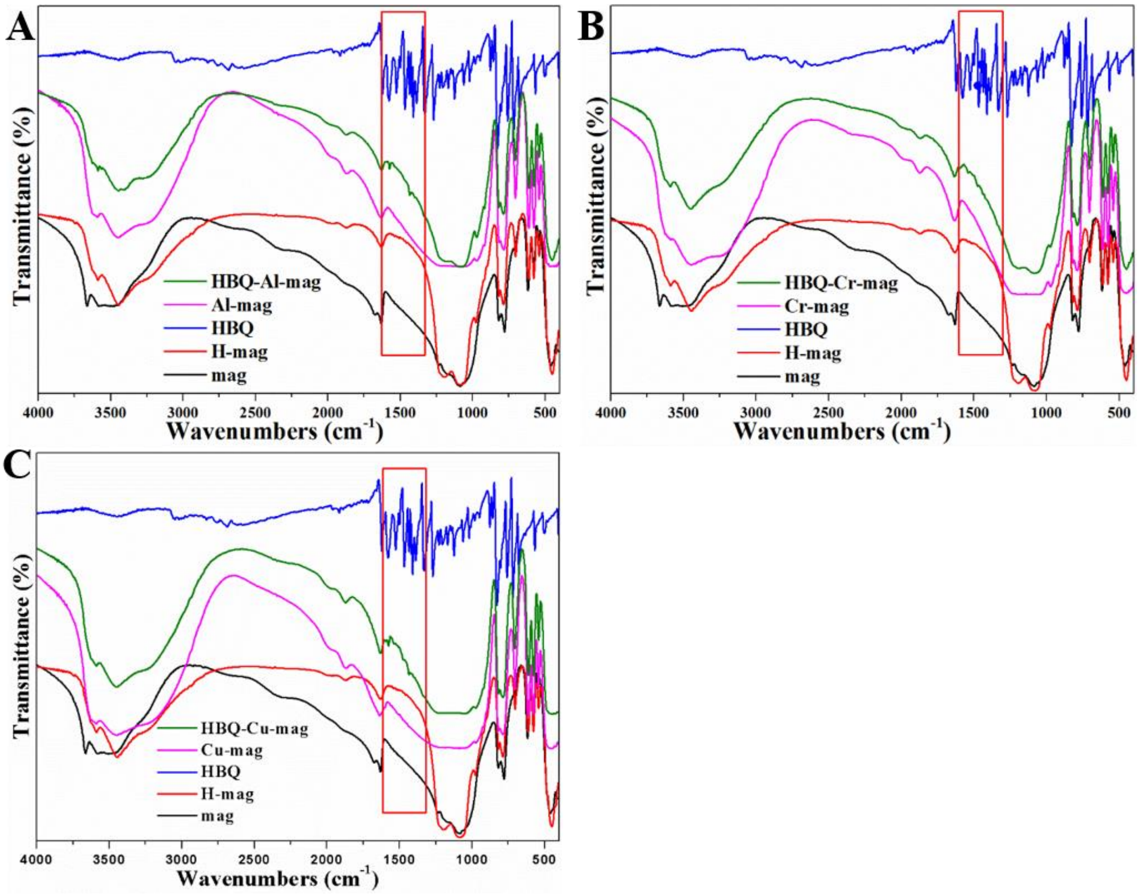

Figure S4. Comparative FTIR spectra of (A) HBQ-Al-mag series, (B) HBQ-Cr-mag series and (C) HBQ-Cu-mag series.

Figure S5

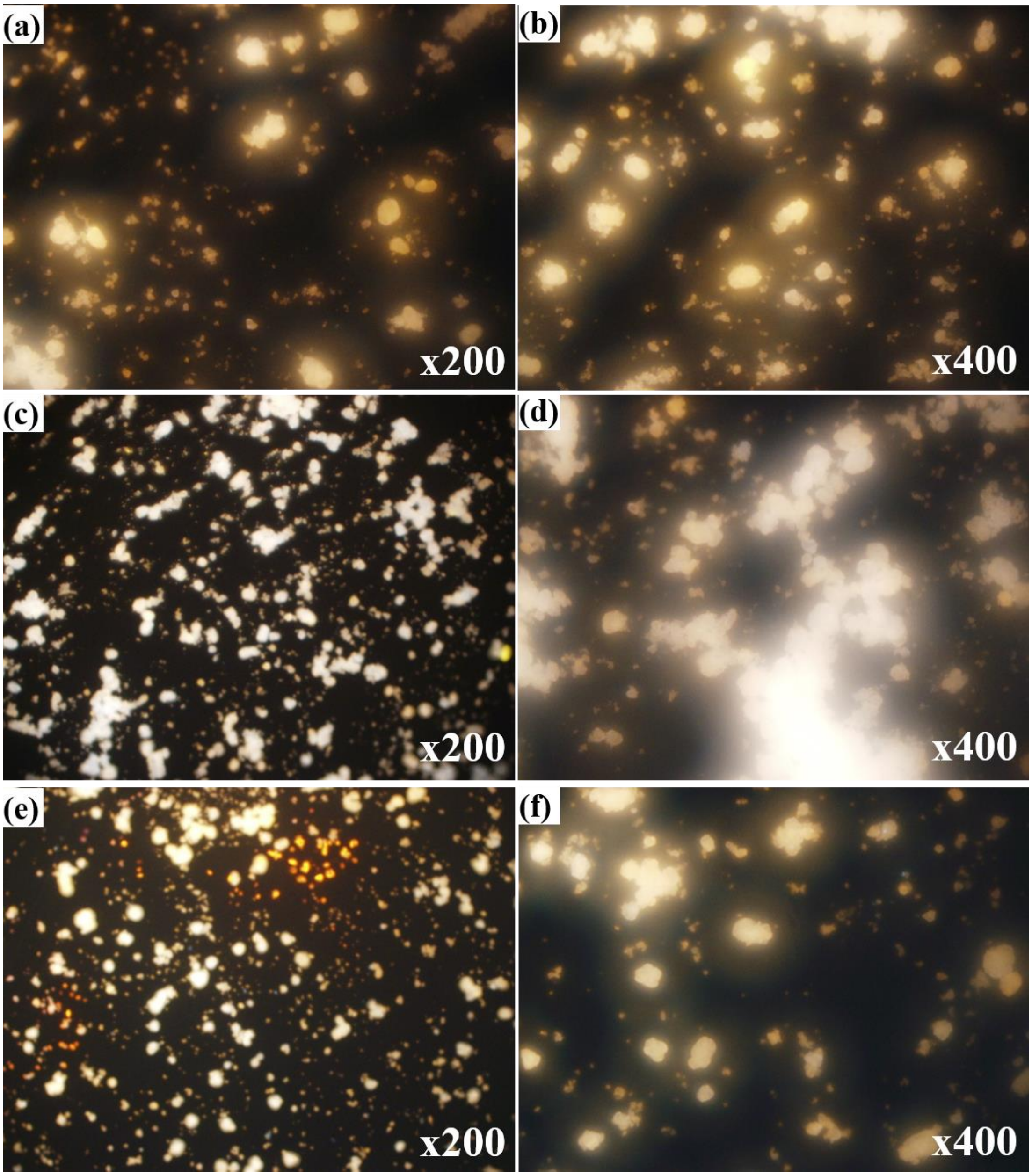

Figure S5. Inverted fluorescence microscope images of HBQ-Al-mag (a-b), HBQ-Cr-mag (c-d) and HBQ-Cu-mag (e-f) under 360 nm UV light irradiation.
